# Dengue and chikungunya: future threats for Northern Europe?

**DOI:** 10.3389/fepid.2024.1342723

**Published:** 2024-01-15

**Authors:** Justine Laverdeur, Daniel Desmecht, Marie-Pierre Hayette, Gilles Darcis

**Affiliations:** ^1^ Department of General Practice, University Hospital of Liège, Liège, Belgium; ^2^ Department of Animal Pathology, Fundamental and Applied Research for Animals & Health, University of Liège, Liège, Belgium; ^3^ Department of Clinical Microbiology, University Hospital of Liège, Liège, Belgium; ^4^ Department of Infectious Diseases and General Internal Medicine, University Hospital of Liège, Liège, Belgium

**Keywords:** one health, Aedes albopictus, chikungunya (CHIKV), dengue (DENV), emerging disease, Northern Europe, arbovirosis

## Abstract

Arthropod-borne viral diseases are likely to be affected by the consequences of climate change with an increase in their distribution and intensity. Among these infectious diseases, chikungunya and dengue viruses are two (re)emergent arboviruses transmitted by *Aedes* species mosquitoes and which have recently demonstrated their capacity for rapid expansion. They most often cause mild diseases, but they can both be associated with complications and severe forms. In Europe, following the establishment of invasive *Aedes* spp, the first outbreaks of autochtonous dengue and chikungunya have already occurred. Northern Europe is currently relatively spared, but climatic projections show that the conditions are permissive for the establishment of *Aedes albopictus* (also known as the tiger mosquito) in the coming decades. It is therefore essential to question and improve the means of surveillance in northern Europe, at the dawn of inevitable future epidemics.

## 1 Introduction

It is now an established fact that the climate is changing as a result of human activities, and that this trend will accelerate in the coming decades, unless we change drastically the way we use energy (1). Consequences on human health are multiple and of extreme importance, and justify a One Health approach that takes global changes into account in public health policies (2, 3).

Among human health domains that are likely to be impacted by climate change, now or in the future, infectious diseases have recently attracted renewed interest. Recent decades have showed a rapid increase of emerging diseases, mainly zoonoses and vector-transmitted diseases (4). Arbovirosis—a group of viral diseases transmitted by arthropod—appear likely to show particular sensitivity to the main abiotic consequences of climate change, namely rising temperature and changing precipitation patterns (5).

Arthropods are affected by external temperature, on which their survival, feeding and reproduction depend (6–8). The alternation of rain and drought, as well as extreme events, favours the development of stagnant water points, and therefore the reproduction of mosquitoes (9, 10). Viral replication within the arthropod vector also depends on the external temperature. Ultimately, the interaction between the vector and the virus results in a bell-shaped temperature response with an optimum, a lower threshold and a higher threshold (10–13).

Evidence of the climate change impact on the distribution of vectors, the endemic area, and the occurrence of outbreaks already exist for many arboviruses, including dengue (14), chikungunya and zika (15), tick-borne encephalitis (16, 17), Crimean-Congo haemorrhagic fever (18) and West Nile virus (5, 19). Current researches are largely focused on the development of predictive distribution models of these diseases, particularly in Europe (20, 21). These models predict an expansion of the endemic areas of arboviruses studied towards higher latitudes and altitudes (22–27). The expected impact is particularly significant in Europe (23, 24, 28). The spread of these pathogens in this immunologically naive population could lead to severe cases and outbreaks (24, 29).

We will focus this review on two emerging *Aedes*-borne arboviruses which are already a threat in some European countries that have to some extent, an animal reservoir and have, according to current models, a predicted expansion in next decades toward northern Europe: chikungunya virus (CHIKV) and dengue virus (DENV).

## 2 Invasive Aedes mosquitoes

*Aedes* (*Ae*.) *spp* mosquitoes, and in particular *Aedes aegypti* and *Aedes albopictus*, are major vectors of arboviruses, among which the yellow fever, dengue, chikungunya and zika viruses (30, 31). Due to their invasion potential and their health importance, they are the subject of entomological surveillance programs at nationals and European level (32, 33).

### 2.1 Aedes albopictus

*Aedes albopictus,* or tiger mosquito, was originally to be found in Asia, but has now spread to all continents (34). Currently, *Ae. albopictus* is introduced in Belgium, and considered established in France and Germany, as well as in large parts of Europe (35, 36). The success of *Ae. albopictus* in our latitudes can be explained by its robust physiology and adaptability. Indeed, this species is capable of surviving and reproducing above the average annual temperature threshold of 10° or 11°, with optimum activity between 25 and 30 degrees, and provided that a minimum of 500 mm of precipitation occurs on an annual basis (37, 38). Even more surprising, European strains of the mosquito show a capacity for hibernation, in the form of eggs which enter diapause in response to the reduction in the photoperiod (37, 39). This diapause allows European strains to withstand temperatures down to −10°C during a short period and increases the hatching rate at the end of winter (39). This species also proliferates in a much more urban environment than its original environment, can feed on a wide range of vertebrates, modify its periods of activity and lay eggs in artificial water points (38, 40). Furthermore, it is a daytime-biter and shows resistance to common insecticides, two characteristics that pose problems in terms of vector control (41).

When comparing the theoretical climatic thresholds of *Ae. albopictus* to current climate data, the fundamental niche of *Ae. albopictus* does not yet include northern parts of Europe, mainly due to insufficient summer temperatures (42). However, more recent studies based on climatic data from areas already colonized by this vector conclude that the fundamental niche is much broader (23, 43). In all cases, the climatic conditions in northern Europe should not represent an obstacle to the establishment (and role of vector) of *Ae. albopictus* by 2050, even in the most favourable climatic scenarios (23, 24, 44).

### 2.2 Aedes aegypti

*Aedes aegypti* was previously established in southern Europe, around the Mediterranean basin, from which it disappeared in the early 1900s (37). In recent years, it has reconquered a small part of European territory, in particular Madeira (45). The relatively high thermal requirements of *Ae. Aegypti* currently prevent its expansion to northern Europe (37). It has an optimum around 29°C and remains capable of proliferating up to 36°C, while the population decreases below the threshold of 15°C (46, 47). This threshold temperature of 15 degrees is also approximately the one below which the adult ceases to be active (48). *Aedes aegypti* is rather anthropophilic, although it can feed on other mammals (37).

The fundamental niche of *Ae. aegypti* does not currently cover northern Europe, unlike some southern regions of France, Italy and Spain (23, 43). *Aedes aegypti* will see its global distribution area increase significantly in the coming decades in every climate change scenario (23, 24, 43). Under the “worst case scenario” hypothesis it could even cease to be a vector in Africa and make a shift towards a northern and more seasonal transmission (24). However, it is not expected for this mosquito to become endemic in northern Europe by the end of the century (23, 43, 49, 50).

### 2.3 Current situation of Aedes aegypti and Ae. albopictus in Europe

Monitoring programs for tropical mosquitoes (in particular *Aedes spp*) aim to see the evolution of their population in Europe. The ECDC distinguishes three phases of colonization of invasive species: the absence, introduction, and establishment of the invasive species. *Ae. albopictus* is already established in most of the France (with the exception of some northern regions), and occasionally leads to autochthonous transmission of arboviruses (51, 52). In Andalusia (Spain), a permanent population of *Ae. aegypti* has been established since the end of the last century, and outbreaks of *Aedes*-transmitted arboviruses have been reported since 2009 (15).

**Figure 1:**
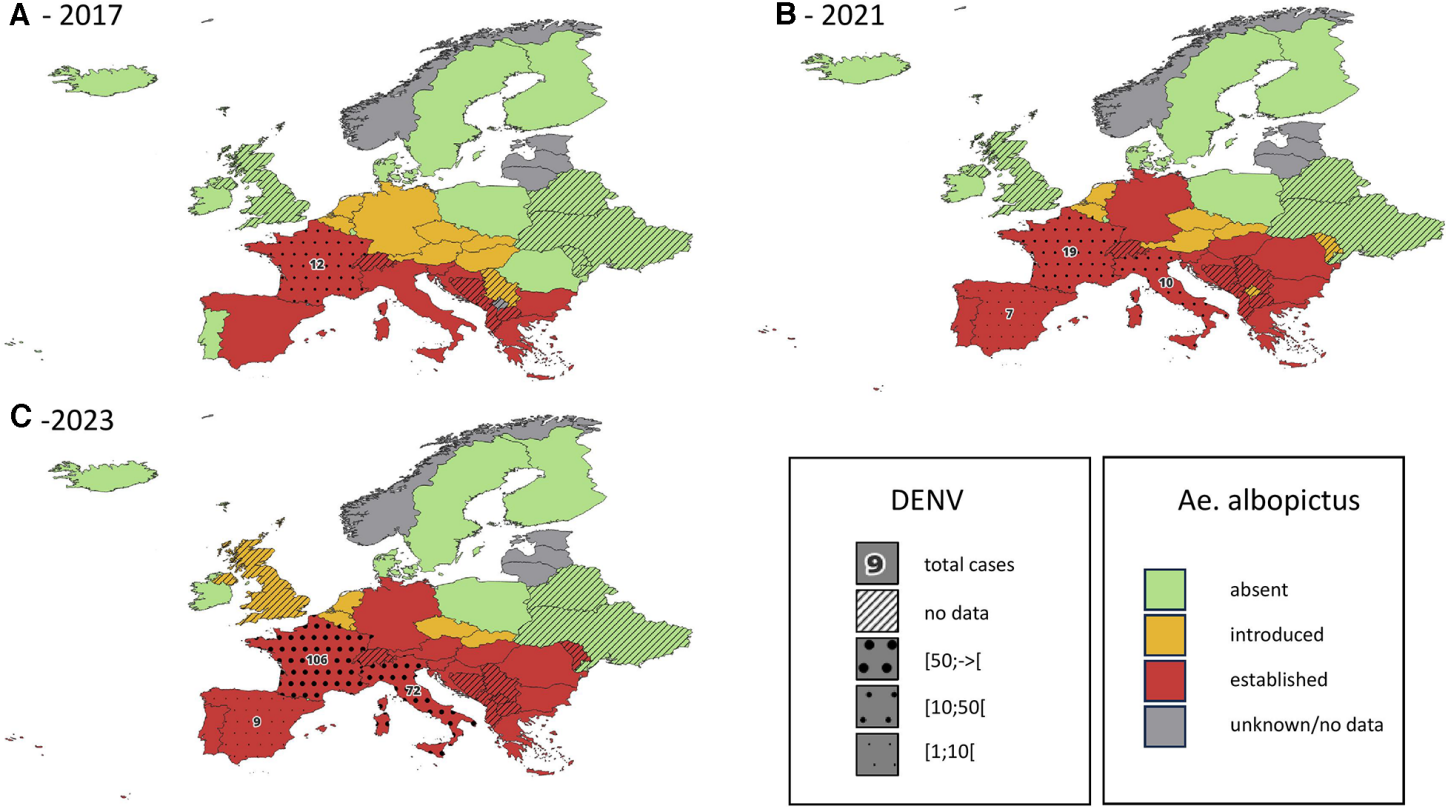
Introduction status of the invasive mosquito species aedes albopictus in Europe, in 2017 (**A**), 2021 (**B**) and 2023 (**C**) cumulative reported autochtonous dengue cases are indicated on the same map for the period 2014-2017 (**A**), 2018-2021 (**B**) and 2022-2023 (**C**) data regarding DENV cases are only available for countries belonging to the EEA. Data obtained from the European Centre for Diseases Prevention and Control (1–4).

*Ae. albopictus* is not yet established (although recently locally introduced) in United Kingdom (35, 53, 54). In the Netherlands, evidence of introduction and even local reproduction of *Aedes aegypti* and *Aedes albopictus* has been found around Schiphol Airport (54, 55). The first specimen of *Ae. albopictus* in Belgium was found in 2000, it is now regularly introduced (36, 56) and some specimens survive the Belgian winter (57). See also Figure 1 for the current and past distributions of Aedes albopictus in mainland Europe.

### 2.4 Other Aedes mosquitoes

*Ae. japonicus* and *Ae. koreica* are other potentially invasive *Aedes* mosquitoes that could cause health issues. There is evidence of competence of *Ae. japonicus* for the transmission of arboviruses such as dengue, chikungunya and Zika virus, in laboratory setting (58, 59). These two mosquitoes are now widely established in Europe (60). Their health importance is unknown to date, and their field transmission competence has not been established in Europe (58, 59).

## 3 Aedes-borne diseases

The main arboviruses transmitted by *Aedes aegypti* (and to varying extents by *Ae. albopictus*) present similar epidemiological features and clinical syndromes.

Most of the time, the natural course of diseases caused by these viruses consists in the succession of a short incubation phase, a febrile phase, and a defervescence phase (61, 62). The defervescence phase progresses within a week either towards recovery or towards a more severe disease (61, 62). Seroconversion (IgM) is generally observed one week after symptoms onset (61). However, cross-reactivity between different flaviviruses can make distinction complicated, particularly in cases of pre-exposure (infection or vaccination) (63). Direct detection by PCR is effective but limited to the first days of the disease (61).

### 3.1 Chikungunya

Chikungunya virus (CHIKV), a togaviridae, is transmitted by *Aedes spp* mosquitoes, especially *Ae. aegypti* and *Ae. albopictus*. Four lineages are currently described, all of which belonging to the same serotype (64). CHIKV was first isolated from a patient in Tanzania in 1952 (65). Its natural cycle, originally described in Asia and the Indian Pacific Islands is sylvatic, with numerous vertebrates (notably non-human primates, bats and ectothermic vertebrates) as reservoirs (66, 67). However, epidemics are characterized by the circulation of the virus in an urban cycle, with a transmission between mosquitoes and humans, the latter serving as reservoirs (66). Occasionally the virus can also be transmitted directly from human to human, through contact with bodily fluids of highly viremic individuals (68).

Chikungunya disease is classically characterized by a sudden onset of high fever, severe arthralgia (often with swelling) and a rash (61, 69). Full recovery is usual within ten days but joint pain can persist in up to 25% of the patients and become chronic (69–71). Severe disease, with neurological, renal, cardiac or metabolic involvement is rare and 1 death per 1,000 cases is observed, mainly among comorbid patients (72).

Chikungunya remained relatively unknown until the early 2000s. In 2004, an epidemic started in Kenya and spread in 2005—2006 to the islands of the Indian Ocean, notably the Reunion Island (66). The epidemic that occurred in Reunion was striking due to its intensity and the fact that its main vector was *Ae. albopictus*, which was previously thought to be only a secondary vector (73). These two characteristics were partially explained by the A226V mutation altering a membrane fusion glycoprotein (74). This mutation makes *Ae. albopictus* slightly more competent than *Ae. aegypti* for the E1-A226V variant, which rapidly constituted the main variant in Reunion (75, 76, 77). Another important cause of the severity of the Reunion epidemic was the immunologically naive nature of the population to this arbovirus (48).

Since then, other significant outbreaks have occurred around the world, including several in Europe. In 2007, the first Chikungunya outbreak in Europe was recorded in Italy (34, 78). In the following years, a few indigenous cases were reported in France, often associated with extreme climatic events (34, 51, 79, 80). If the CHIKV strain with the E1-A226V mutation shows, in most studies, better transmissibility by *Ae. albopictus*, it is not the only strain transmitted in Europe by this vector (81). Indeed, a strain without this mutation caused an epidemic in Italy in 2017 (82), during which the competence of *Ae. albopictus* was similar to the 2007 CHIKV-E1-A226V outbreak (83).

As discussed previously, the competent vector *Ae. albopictus* is already found in northern Europe. Furthermore, the incubation of CHIKV in *Ae. albopictus* can occur at temperatures as low as 18°C, resulting in a high transmission rate (84). Therefore, the climate in some parts of northern Europe is already conducive to a Chikungunya outbreak in summer (25, 26). In the coming decades, temperature conditions are expected to allow the transmission of Chikungunya in northern Europe for 3 months per year (85).

### 3.2 Dengue

Dengue virus (DENV), a flavivirideae, was first isolated in 1943 (86). There are four serotypes, initially isolated in Asia and Oceania, which now circulate widely throughout the world, mainly in Asia, Africa and America (87, 90). Its enzootic cycle involves specific sylvatic strains circulating between non-human primates and arboreal mosquitoes (sylvatic cycle). On the other hand, the epidemic circulation of urban DENV (urban cycle) is classically described as exclusively depending on the human host and (peri-)urban mosquitoes' vector *Aedes aegypti* and *Ae. Albopictus* (86). However, spillovers from the enzootic cycle are regularly documented and four of these cross-over species transmission events are thought to be the origin of the four current urban serotypes of dengue (88). Moreover, there is recent evidence supporting the fact that other vertebrates can be infected and their role as urban reservoirs can't be ruled out (89). These findings suggest that the role of the animal-human interface in the present and future (re)emergence of dengue shouldn't be overlooked.

The disease caused by this virus is dengue fever, a usually mild disease with a spontaneous favourable evolution within 1–2 weeks (90). However, a more serious disease is also observed, severe dengue, preferentially affecting children before the age of 15, and fatal in 20% of cases in the absence of appropriate supporting care (91). Severe dengue occurs preferentially in areas of coexistence of different subtypes, and the main causal mechanism is linked to sequential infection by two different subtypes, the previous antibodies forming antibody-virus complexes during the next infection (92). However, the antibody not being specific to the subtype fails to inactivate the virus and leads to the antibody-dependent enhancement of the viral replication, increasing the risk of sepsis (92).

Dengue has undergone the most dramatic increase among infectious diseases during the last 50 years, with a 30-fold increase (93). Evidence already exists in Asia of the impact of climate change on its distribution (14). Models predict an increase in the endemic area of ​​dengue, an extension of the transmission season and a shift toward a younger age at secondary infection by dengue under the effect of climate change (27, 94).

Although *Ae. aegypti* is historically considered the main vector of dengue, the role of *Aedes albopictus* in the transmission of dengue is increasingly recognized. Its involvement in previous epidemics in temperate zones has been proven (95). Some studies even consider that its robustness and lifespan, make *Ae. albopictus* a more competent vector than *Ae. aegypti* for DENV (96). The optimum temperature for dengue transmission depends on the vector concerned (97). *Ae. albopictus* is competent for dengue transmission at temperatures as low as 21°C (with an optimum around 30°C) (98). In this interval, increasing temperature decreases the incubation period and increases the rate of transmission (99).

In mainland France where *Ae. albopictus* is established, autochthonous transmission of DENV has been observed since 2010 (51, 100, 101). This transmission mode remains confined to small self-limited outbreaks of a few individuals, even if 2022 summer has seen a dramatic increase of these events with 65 indigenous cases (more than the total from 2010 to 2021) (102, 103). Figure 1 shows the cumulative reported autochthonous dengue cases in countries of the European Union, in parallel with the distribution of Ae. albopictus.

Given the projections regarding the likely establishment of *Ae. albopictus* in the short term in northern Europe, the rather moderate temperatures allowing transmission of dengue by this mosquito, observations already reported in neighboring countries, and inevitable introductions of DENV (via infected people), it therefore seems probable that dengue epidemics will occur in the coming decades in northern Europe.

## 4 Discussion

Aedine mosquitoes, and in particular *Aedes albopictus*, have shown a recent large geographical expansion, becoming established in regions ever further north of the European continent. *Ae. albopictus* is considered as one of the more threatening invasive species in the world (104). Even under current climatic conditions, it is expected that the mosquito will continue its dissemination across the European continent in the next years through human-mediated spread, considering its fundamental niche already includes most of this territory (23, 49). Afterwards, as the suitability of European climate for this mosquito is expected to continually increase in the next decades, its distribution will likely include northern Europe by the end of the century (49).

Both *Ae. albopictus* and *Ae. aegypti* are vectors of human health threatening viruses, among which dengue and chikungunya are of particular concern. Data from other regions of the world show that the first epidemics of *Aedes*-borne viruses were observed between 5 and 15 years after the establishment of *Ae. albopictus* (49). On the American continent, the existence of competent vectors for ZIKV and CHIKV allowed the introduction of these two viruses in the 2010s. It resulted in large epidemics in the following years and ultimately the endemic establishment of these diseases (105, 106). The first European data tend to confirm this pattern, with an important increase of autochthonous DENV cases in countries where Ae. albopictus has been well established for several years (see Figure 1).

These data converge to demonstrate the probability that large arboviruses epidemics could occur in northern Europe in next decades. They also tend to support the need to act on different levels to prevent these arboviruses from posing a serious public health threat in the coming decades.

Entomological surveillance of invasive mosquitoes species already exists and is coordinated at European level (107). However, on the field, some disparities exist in the degree and methods of surveillance around high-risk introduction routes (54). Resources to strengthen and improve coordination of entomological surveillance could therefore help to prevent the dissemination, especially of *Ae. albopictus*, in the already climatically suitable European regions. Furthermore, several initiatives have been taken to locally control the mosquito population, including eradication programs for *Ae. aegypti* in the Mediterranean (92) or the use of larvicides to control introduced populations in Belgium (36).

Despite the efforts put in the surveillance and control of invasive mosquitoes, it is likely that *Ae. albopictus* will finally become established in large parts of Europe, and some resources should be allocated to the next steps of the risk mitigation (49). One of these next steps have to be the increase of knowledge and awareness concerning arboviral diseases among healthcare workers (108, 109). Furthermore, an epidemiologic monitoring as soon as the vector is established could be effectively done by an entomo-virological surveillance followed by serologic surveys (110). Serological surveys are useful to monitor the level of viral circulation and take timely mitigation measures (111). They also show that, among travellers, the circulation of dengue is underestimated (112). However, the serological diagnosis of arboviruses is still a challenge in many ways, due to laboratory lack of preparedness and the technical challenge posed by cross-reactivity among arboviruses (113, 114). Efforts to improve laboratory preparedness, physician awareness and to develop accurate and cost-effective diagnostic tools would therefore help assess the current circulation of these arboviruses and prepare to respond in a timely manner to a future outbreak.

Finally, when basing assumptions on climate change modeling scenarios, it must not be neglected that the more unfavorable the mitigation scenario, the greater the uncertainty regarding temperature increases and climate extremes (1). Preparing for the inevitable consequences of the climate change should therefore not make us forget the crucial importance of limiting its (potentially unpredictable) impact by continuing efforts to reduce greenhouse gases and mitigate other human-mediated global changes.
